# Titanium–Aluminum–Vanadium Surfaces Generated Using Sequential Nanosecond and Femtosecond Laser Etching Provide Osteogenic Nanotopography on Additively Manufactured Implants

**DOI:** 10.3390/biomimetics10080507

**Published:** 2025-08-04

**Authors:** Jonathan T. Dillon, David J. Cohen, Scott McLean, Haibo Fan, Barbara D. Boyan, Zvi Schwartz

**Affiliations:** 1 Department of Biomedical Engineering, Virginia Commonwealth University, Richmond, VA 23284, USA; dillonj4@vcu.edu (J.T.D.); djcohen@vcu.edu (D.J.C.); bboyan@vcu.edu (B.D.B.); 2 Spine Wave, Inc., Shelton, CT 06484, USA; smclean@spinewave.com (S.M.); hfan@spinewave.com (H.F.)

**Keywords:** titanium, additive manufacturing, femtosecond laser, implant surfaces, bone marrow stromal cells, MSCs, osseointegration

## Abstract

Titanium–aluminum–vanadium (Ti6Al4V) is a material chosen for spine, orthopedic, and dental implants due to its combination of desirable mechanical and biological properties. Lasers have been used to modify metal surfaces, enabling the generation of a surface on Ti6Al4V with distinct micro- and nano-scale structures. Studies indicate that topography with micro/nano features of osteoclast resorption pits causes bone marrow stromal cells (MSCs) and osteoprogenitor cells to favor differentiation into an osteoblastic phenotype. This study examined whether the biological response of human MSCs to Ti6Al4V surfaces is sensitive to laser treatment-controlled micro/nano-topography. First, 15 mm diameter Ti6Al4V discs (Spine Wave Inc., Shelton, CT, USA) were either machined (M) or additively manufactured (AM). Surface treatments included no laser treatment (NT), nanosecond laser (Ns), femtosecond laser (Fs), or nanosecond followed by femtosecond laser (Ns+Fs). Surface wettability, roughness, and surface chemistry were determined using sessile drop contact angle, laser confocal microscopy, X-ray photoelectron spectroscopy (XPS), and scanning electron microscopy (SEM). Human MSCs were cultured in growth media on tissue culture polystyrene (TCPS) or test surfaces. On day 7, the levels of osteocalcin (OCN), osteopontin (OPN), osteoprotegerin (OPG), and vascular endothelial growth factor 165 (VEGF) in the conditioned media were measured. M NT, Fs, and Ns+Fs surfaces were hydrophilic; Ns was hydrophobic. AM NT and Fs surfaces were hydrophilic; AM Ns and Ns+Fs were hydrophobic. Roughness (Sa and Sz) increased after Ns and Ns+Fs treatment for both M and AM disks. All surfaces primarily consisted of oxygen, titanium, and carbon; Fs had increased levels of aluminum for both M and AM. SEM images showed that M NT discs had a smooth surface, whereas AM surfaces appeared rough at a higher magnification. Fs surfaces had a similar morphology to their respective NT disc at low magnification, but higher magnification revealed nano-scale bumps not seen on NT surfaces. AM Fs surfaces also had regular interval ridges that were not seen on non-femto laser-ablated surfaces. Surface roughness was increased on M and AM Ns and Ns+Fs disks compared to NT and Fs disks. OCN was enhanced, and DNA was reduced on Ns and Ns+Fs, with no difference between them. OPN, OPG, and VEGF levels for laser-treated M surfaces were unchanged compared to NT, apart from an increase in OPG on Fs. MSCs grown on AM Ns and Ns+Fs surfaces had increased levels of OCN per DNA. These results indicate that MSCs cultured on AM Ns and AM Ns+Fs surfaces, which exhibited unique roughness at the microscale and nanoscale, had enhanced differentiation to an osteoblastic phenotype. The laser treatments of the surface mediated this enhancement of MSC differentiation and warrant further clinical investigation.

## 1. Introduction

Titanium alloy implants, specifically those composed of titanium–aluminum–vanadium (Ti6Al4V ELI), are widely used in orthopedic and dental applications due to their exceptional mechanical properties, corrosion resistance, and biocompatibility [1,2,3]. The long-term success of these implants depends heavily on the promotion of osteogenesis and successful osseointegration, which is the direct and functional connection between the load-bearing implant and living bone. Despite the favorable characteristics of Ti6Al4V implants, osseointegration is not always achieved, particularly in patients with clinical conditions that impact osteogenesis. Surface modifications have thus been employed to enhance biological response and promote better long-term patient outcomes [4]. Traditionally, surface modifications such as sandblasting and acid etching have been widely used to improve osseointegration; however, they offer limited control over surface topography. In contrast, laser processing provides precise modification of micro- and nanoscale surface features, allowing the creation of surfaces that more closely mimic natural bone topography [5,6,7]. Studies have demonstrated that laser-modified titanium implant surfaces exhibit improved osteogenic activity in vitro due to increased roughness at the micro- and nanoscale and the formation of laser-induced periodic surface structures (LIPSS) [8]. Additionally, laser surface modification facilitates the deposition of bioactive coatings such as hydroxyapatite, further enhancing implant osseointegration [9]. These advancements in surface modification technology suggest that laser-etched Ti6Al4V implants have the potential to reduce implant failure significantly.

Research into laser modification of Ti6Al4V implants has shown promising results in producing a surface with morphology that favors osseointegration and improved patient outcomes [3,10,11,12,13]. The mimicking of not only the micro-scale roughness but also the nanometer topography naturally present in bone tissue is a goal of many surface modifications [14,15]. Specifically, recent studies indicate that surface morphology mimicking osteoclast resorption pits at the micro- and nano-scale promotes differentiation of bone marrow stromal cells (MSCs) and osteoprogenitor cells to an osteoblastic phenotype [16]. The interaction of the cells with these modified surfaces activates key signaling pathways, including the increased production of osteocalcin, osteoprotegerin, and vascular endothelial growth factor [17]. In addition, MSCs grown on roughened implant surfaces (Sa 1.8) showed increased levels of anti-inflammatory interleukins and cytokines and decreased levels of proinflammatory interleukins and cytokines compared to smooth (Sa 0.5) surfaces [18,19]. These findings point toward the potential of laser surface modifications to generate highly reproducible implants with improved patient recovery time and reduced implant failures [20,21,22].

Different parameters of the laser used in the ablation can greatly impact the qualities of the resulting surface [23]. Nanosecond lasers, with longer pulse durations relative to femtosecond lasers, have much greater thermal effects during laser–substrate interaction. Localized melting and vaporization generate micro-roughened and resolidified features that can promote bone cell adhesion [24]. The shorter pulse duration of femtosecond lasers leads to less thermal diffusion and has been found to produce highly precise, nano-scale resolution surface features while limiting the loss of the material’s bulk properties [25,26].

Additive manufacturing (AM) techniques offer advantages for dental and orthopedic implant manufacturing, as they enable the fabrication of complex geometries and surface morphology. Furthermore, AM allows control over macro- and micro-scale surface architecture, which can influence cell behavior and bone healing. Recent studies have demonstrated how this control is achieved through the tunability of printing parameters [27]. In AM metal and metal alloy platforms, such as Ti6Al4V, surface roughness and chemistry can be further tailored through post-processing methods such as laser ablation to improve osseointegration.

The aim of the present study was to evaluate the osteogenic and immunomodulatory effects of surfaces generated by nanosecond and femtosecond lasers on MSC differentiation in vitro. While there is a large body of research on the modification of machined and additively manufactured metal surfaces using these two different lasers, less is known about how the resulting surface properties impact biological outcomes. Ti6Al4V implants are fabricated using additive manufacturing and traditional machining, but how differences in the physical and chemical properties of the resulting implant surface might alter the micro/nanotopography achieved by laser treatment and the downstream effects on MSC differentiation are unknown. These are important considerations in designing implants for use in bone. Recent studies indicate that Ti6Al4V implants fabricated by additive manufacturing have a lower elastic modulus compared to traditionally manufactured implants [28], and a lower modulus is favored for implants in orthopedic and dental applications, as it can better match that of natural bone, limiting stress shielding that can cause bone resorption and implant loosening [29,30].

To explore how manufacturing methods impact the topographies achieved by laser treatment and the responses of MSCs to the resulting surface, we examined the effect of sequential nanosecond and femtosecond laser ablation compared to individual treatments. This research was carried out with an in vitro model that uses growth media (GM) rather than osteogenic media (OM) to culture the MSCs, thereby eliminating any artifact of the OM additives, including dexamethasone or beta-glycerophosphate (BGP), on the results. Thus, results showing MSCs shift to a more osteoblastic phenotype can be attributed to the micro/nano-scale features of the implant surface and not the dystrophic calcification as a result of the cells being grown in OM [19].

## 2. Materials and Methods

### 2.1. Disk Production

Laser-ablated disks were fabricated in three steps: (1) machining or laser powder additive manufacturing (3D printing) from stock raw material; (2) laser ablation to incorporate intended micro/nano topologies on disk surfaces; and (3) terminal cleaning, packaging, and sterilization. Disks without laser ablation (as-machined or as-3D printed) were controls (NT) for all experiments. Machined disks were manufactured with a traditional subtractive milling and subsequent blasting finishing process using implant-grade Ti6Al4V ELI conforming to ASTM F136. All materials and processes were identical to the typical machined titanium implant manufacturing process. Then, 3D-printed disks were additively manufactured via direct metal laser sintering (DMLS) from implant-grade titanium alloy powder using a ProX DMP 320 3D-Printer system (3D Systems, Rock Hill, South Carolina), followed by hot isostatic pressing (HIP) and finishing of the manufactured coupons. The Ti6Al4V powder material conformed to ASTM F3001. All materials and processes were identical to typical titanium alloy implants’ materials and additive manufacturing processes.

All disks were 1.5 cm in diameter. Machined (M) disks were 2 mm in height, and additively manufactured (AM) disks were 5 mm in height. Surface treatments included no laser treatment (NT), nanosecond laser (Ns), femtosecond laser (Fs), or nanosecond followed by femtosecond laser (Ns+Fs). They were processed, terminally cleaned, packaged, and gamma-sterilized using the same methods as those used for clinically available titanium alloy implants (Spine Wave, Inc.).

### 2.2. Laser Treatment

The laser ablation system used for this study was the GF laser system P400U (Precision Coatings, Smithfield, RI, USA), with nanosecond and femtosecond laser sources. The nanosecond source was a ytterbium fiber laser (IGP Photonics, Marlborough, MA, USA) with a central wavelength of 1064 nm, a pulse duration of 200 nanoseconds, a dosing speed of 2000 m/s, a power of 100%, and a Hatching distance of 0.02 mm. These parameters were based on an industrial laser texturing process designed to enhance grip on hand tools, with reduced energy to minimize the heat effect on the base material while maintaining the desirable roughness and pit/pore size. The process resulted in a carbon film on the surface, which was removed using an industrial cleaning protocol that leveraged the heating properties of the femtosecond laser. The femtosecond source was a Yb: YAG crystal laser with a femtosecond wavelength source central wavelength of 1030 nm, a pulse duration of 295 femtoseconds, a dosing speed of 2000 m/s, a power of 70%, and a Hatching distance of 0.02 mm.

### 2.3. Surface Laser Treatment Verification

Only one side of each disk received laser treatment. To identify the “laser-treated” (T) side of each disk, the Spectro 1 handheld spectrophotometer (Variable Company, Winston-Salem, NC, USA) was used. Once connected to the Spectro application, the “Quick Compare” function was opened. Disks with two untreated sides (NT) were used as controls. Lighting (L) and the change in color variable (ΔE) readings were compared for each sample, and the T side was determined to be the side with the different L reading and higher ΔE. All surface characterization and in vitro testing were performed after it was verified using this method that the T side was being tested, and not the NT side.

### 2.4. Surface Characterization

Gamma-irradiated surfaces were removed from their sterile packaging and immediately tested for either contact angle, surface chemistry, or roughness.

#### 2.4.1. Scanning Electron Microscopy

Surface topography and morphology were qualitatively visualized using scanning electron microscopy (SEM, Hitachi SU-70, Tokyo, Japan). Samples were affixed to SEM imaging mounts with carbon conductive adhesive tape. Surfaces were imaged at a working distance of 5 mm under an ion current of 56 μA and an acceleration voltage of 5 kV. Each group was imaged at six different locations of a single surface.

#### 2.4.2. Contact Angle Analysis

Surface wettability was determined by performing sessile drop tests using a goniometer (Model 250 Goniometer, Ramé-Hart Instrument Co., Succasunna, NJ, USA). Drops of 3 μL of room temperature DI water were placed on leveled titanium surfaces. An image of the drop was taken immediately and then every 5 s thereafter for a total of 15 s, resulting in 4 images per drop. The right and left angles at each time point were averaged to obtain a single value for each drop. This was performed at six different locations on a single surface of each group to control variability on each surface.

#### 2.4.3. Roughness Analysis

Confocal surface profilometry was performed using a confocal laser scanning microscope (Zeiss LSM 710, Carl-Zeiss AG, Oberkochen, Germany) equipped with Zen 2.3 (black edition) software. Roughness readings were obtained through the Zeiss Plan-APOCHROMAT 20×/0.8 objective and T80/R20 reflected visible light main beam splitter. The 405 nm laser employed was set to 2.0% power. A pinhole of 17.2 μm and a master gain of 500 AU were used. The z-stack step size was 0.62 μm, and the surface of 425.1 × 425.1 μm images had a 0.42 μm pixel size. The final topographical images had a Gaussian filter set to 3 pixels to reduce noise. Roughness values Sa (arithmetic mean deviation) and Sz (maximum peak to valley distance) are topographical roughness parameters averaged across the entire 425.1 μm^2^ measurement area using the Zen surface roughness measurement tools. Final Sa and Sz values were obtained from averaging measurements taken at six different locations for a single surface per group. It was found that interpolation of non-measured points was necessary due to the effects of shadowing in the roughest surfaces. This was performed for all surfaces using the incorporated feature in Zen software. In addition, a 20 μm high-pass filter was applied to the z-stack projections in order to filter out a portion of the larger-scale topographical roughness and gain insight into the roughness features that pertain to bone marrow stromal cells (~20 μm in length).

#### 2.4.4. Surface Chemistry

Surface chemistry was analyzed using X-ray photoelectron spectroscopy (XPS) (PHI VersaProbe III Scanning XPS, Physical Electronics Inc., Chanhassen, MN, USA). Sterile surfaces were secured to mounts using copper clamps. Mounts and clamps were first cleaned in a sonication bath submerged in an ethanol solution for 1 min. The X-ray source settings were a 100 μm beam size, 50 W power, and 15 kV beam energy. Survey spectra were obtained using a pass energy of 280 eV. A total of 6 different 500 μm^2^ areas on a single surface from each group were scanned. Charge correction for all data was set to C-C bonds at 248.4 eV.

### 2.5. Cell Culture

Commercially procured passage 2 human bone marrow stromal cells (MSCs) were obtained from a 23-year-old white female (Ossium Health, San Francisco, CA, USA). The provider tested the cells before purchase for positive expression of CD73, CD90, and CD105. Cells were also tested negative for hematopoietic and HLA class II markers. Cells were cultured on tissue culture polystyrene (TCPS) T-flasks in growth media (GM) consisting of αMEM without nucleotides, 4 μM of L-glutamine, 10% heat-inactivated fetal bovine serum, 100 U/mL of penicillin, and 100 μg/mL streptomycin. The flasks were incubated in 100% humidity, 5% CO_2_, and 37 °C, and subcultured at 80% confluence. When they reached 80% confluence in passage 4, they were subcultured onto the Ti6Al4V disks. These cells show the ability to differentiate and show an osteoblast phenotype on a microrough surface or after culturing them on TCPS in osteogenic media. For all in vitro experiments, cells were plated at 9500 cells/well on a TCPS 24-well plate containing the different Ti6Al4V surfaces. A TCPS group was plated as an optical control. Cells were given fresh GM after 24 h of incubation, then every 48 h thereafter. On day 7, cells were given fresh GM and incubated for 24 h before the conditioned media were harvested and stored immediately at −80 °C for subsequent analysis. The cell layers were then briefly rinsed twice with sterile 1X PBS and lysed with 0.5 mL/well 0.05% Triton X-100. Cell layer lysates were immediately stored at −80 °C for subsequent analysis.

### 2.6. Cellular Response

Before analysis, cell layer lysate samples were thawed and sonicated at 40 V for 5 s/well (VCX 130; Vibra-Cell, Newtown, CT, USA). Total DNA content was obtained using the fluorescence-based QuantiFluor dsDNA kit (Promega, Madison, WI, USA).

Thawed conditioned media samples were used to quantify the bone proteins osteocalcin, osteopontin, and osteoprotegerin, as well as the vascularization marker vascular endothelial growth factor 165 (VEGF). The standard procedures provided with the enzyme-linked immunosorbent assay (ELISA) kits (R&D Systems, Inc., Minneapolis, MN, USA) were followed. Cell number reflects a combination of cell attachment, proliferation, and death. Our goal was to compare the effects of the surface on the cultures at the time of harvest. Without determining the specific contributions of each parameter to the outcome, we normalized the data to the final cell number. The amount of DNA per cell is a relatively constant value, so we used it as an indication of the number of cells present in the culture at the time of harvest, irrespective of how that number was achieved. Enzyme activity was normalized to the protein content of the cell layer, which provides information on the relative rate at which the enzyme does its job (specific activity). This measurement is independent of the number of cells, but gives an indication of how the cells are functioning. All colorimetric assays and ELISAs were quantified using the Synergy H1 (BioTek Instruments, Winooski, VT, USA) microplate reader.

### 2.7. Statistical Analysis

All the surface characterization data were analyzed as the means ± standard error of 2 disks. Cell response data were analyzed as the means ± standard error of six independent cultures per variable per experiment. Each experiment was repeated a minimum of three times to ensure the reliability of the results, and the treatment/control ratios from the repeated experiments were compared. One-way analysis of variance (ANOVA) with multiple comparisons between groups using a two-tailed Tukey’s post hoc test was used for statistical analysis. Statistical significance was considered for results with a *p*-value less than 0.05. All statistical analyses were performed using GraphPad Prism v10.02 software.

## 3. Results

**Figure 1:**
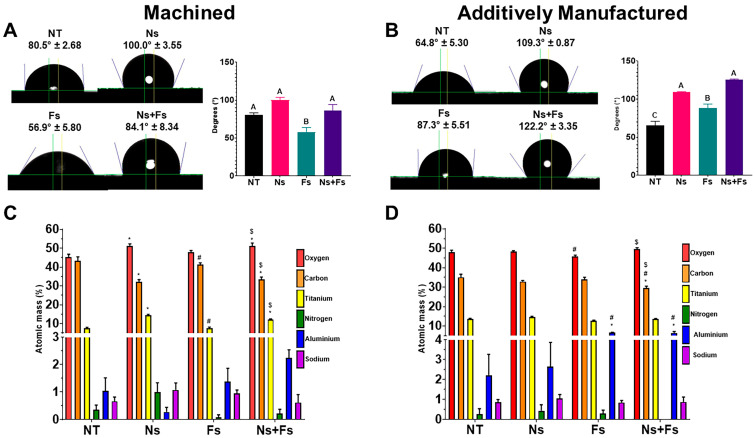
Laser ablation effects on surface properties. Sessile water droplet test of machined surfaces (**A**) and additively manufactured surfaces (**B**). X-ray photoelectron spectroscopy to assess concentrations of elements present on the surface of machined surfaces (**C**) and additively manufactured surfaces (**D**). Surface treatments included no laser treatment (NT), nanosecond laser (Ns), femtosecond laser (Fs), or nanosecond followed by femtosecond laser (Ns+Fs). For (**A**,**B**), groups not sharing a letter are significantly different (*p* < 0.05). For (**C**,**D**), * indicates significance (*p* < 0.05) vs. NT for that element; # indicates significance (*p* < 0.05) v. Ns for that element; $ indicates significance (*p* < 0.05) v. Fs for that element. Data are means +/− SEM (*n* = 6).

Surface wettability varied with the treatment method. Machined surfaces that were not laser-treated were hydrophilic (Figure 1A). Although both Fs and Ns+Fs were also hydrophilic by definition (having a contact angle below 90°), only the contact angle of the Fs group was significantly decreased compared to the other groups.

Ns laser treatment caused the machined surfaces to become hydrophobic (having a contact angle greater than 90°). For additively manufactured disks, the NT and Fs surfaces were hydrophilic (Figure 1B). The Ns treatment caused the surfaces to be hydrophobic, even after treatment with Fs. Ns treatment of machined surfaces exhibited higher O and Ti but lower C than the NT surfaces (Figure 1C). Fs treatment increased surface Al compared to NT and NT treated first with nanosecond laser (Figure 1D). Carbon detected on the Ns+Fs treated surfaces was decreased compared to all other groups.

**Figure 2:**
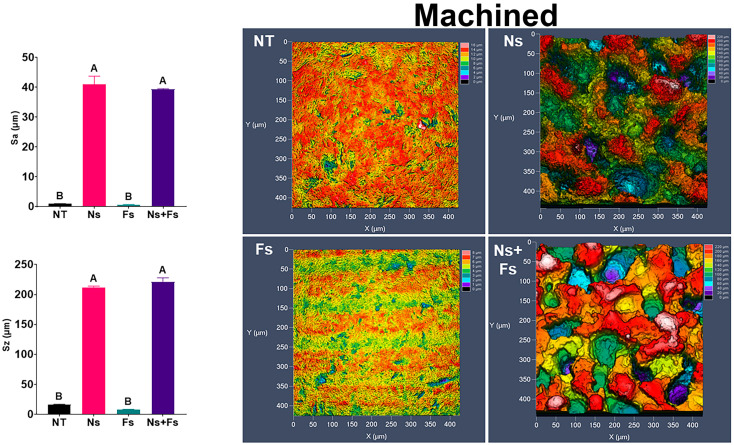
Surface roughness and confocal LSM microscopy demonstrating the topographical differences due to various laser ablation treatments on machined surfaces. Surface treatments included no laser treatment (NT), nanosecond laser (Ns), femtosecond laser (Fs), or nanosecond followed by femtosecond laser (Ns+Fs). Groups not sharing a letter are significantly different (*p* < 0.05). Data are means +/− SEM (*n* = 6).

**Figure 3:**
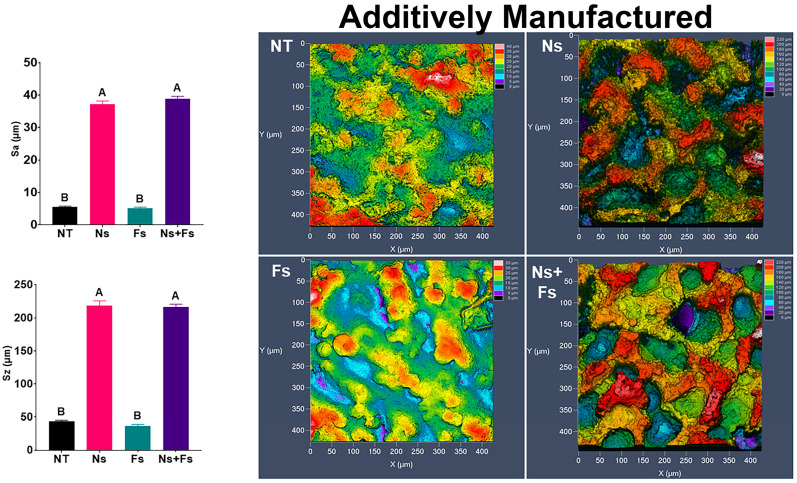
Surface roughness and confocal LSM microscopy demonstrate the topographical differences due to various laser ablation treatments on additively manufactured surfaces. Surface treatments included no laser treatment (NT), nanosecond laser (Ns), femtosecond laser (Fs), or nanosecond followed by femtosecond laser (Ns+Fs). Groups not sharing a letter are significantly different (*p* < 0.05). Data are means +/− SEM (*n* = 6).

Roughness was also modified by laser treatment. Both machined and additively manufactured surfaces exhibited significant increases in arithmetical mean deviation (Sa) and maximum peak-to-valley roughness (Sz) values when treated with the nanosecond laser, which were not altered by further treatment with the femtosecond laser (Figure 2 and Figure 3). These findings are consistent with what is seen on the confocal LSM images of the surfaces. In both groups with Ns treatment, the fluctuations in surface topography were increased. However, some differences were associated with the fabrication method used. Confocal LSM imaging of the machined surfaces revealed a repeating ridge pattern in the femtosecond laser-treated surface that was not found on the additively manufactured surfaces.

To better understand the topographical difference experienced by an hMSC, which is roughly 20 μm in length, Sa and Sz calculations were repeated on LSM images with a 20 μm high-pass filter. The profilometry (Supplementary Figure S1) and Sa and Sz (Supplementary Figure S2) were analyzed from these images. The femtosecond laser ablation significantly reduced the Sa compared to the nanosecond-only laser-treated surface in both machined and additively manufactured disks.

**Figure 4:**
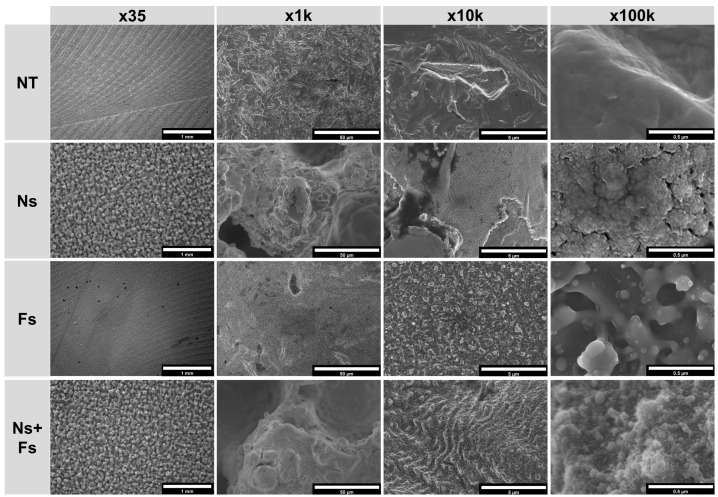
Scanning electron micrographs of machined surfaces showing the generated micro- and nano-scale morphological differences between laser treatments at various magnifications. Surface treatments included no laser treatment (NT), nanosecond laser (Ns), femtosecond laser (Fs), or nanosecond followed by femtosecond laser (Ns+Fs).

**Figure 5:**
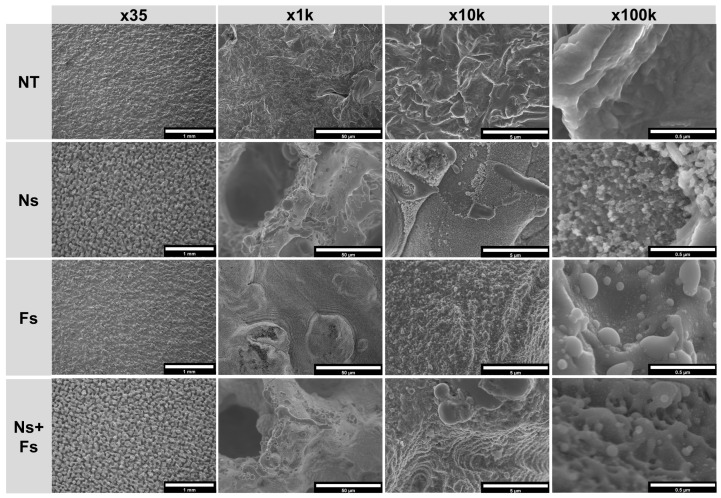
Scanning electron micrographs of additively manufactured surfaces showing the generated micro- and nano-scale morphological differences between laser treatments at various magnifications. Surface treatments included no laser treatment (NT), nanosecond laser (Ns), femtosecond laser (Fs), or nanosecond followed by femtosecond laser (Ns+Fs).

The SEM analysis indicated that the NT surfaces were homogeneous and differed depending on the manufacturing method (Figure 4 and Figure 5). The effects of the laser treatments were also homogeneous across the disk surface. In the lower-magnification SEM micrographs of the machined surfaces, the increased roughness caused by the nanosecond laser was evident on the Ns and Ns+Fs surfaces compared to the disks treated with the femtosecond laser, which caused a wave-like pattern likely from the machining process (Figure 4). In contrast, the nanosecond laser caused a grain-like pattern. At the higher magnifications, this appeared to have a nanoscale cauliflower-like morphology. The Fs surfaces at high magnification had nanoscale evidence of rapidly melting and solidified material, and in the Ns+Fs micrographs at high resolution, these characteristics combined with an irregular wave-like pattern for the structure.

The increased roughness from the additive manufacturing process was evident in the low-magnification SEMs of the additively manufactured surfaces (Figure 5). The grain-like pattern noted on the machined disks was also present on the Ns and Ns+Fs treated AM disks, and at higher magnification, the Ns surfaces had a similar nanoscale grain structure, which was observed, to a lesser extent, in the Ns+Fs group. The Fs surface had the rapid melting and solidifying-produced morphology found on the machined surfaces. These spherical nanoscale structures were also present in the Ns+Fs group, where a wave-like structure was also present.

**Figure 6:**
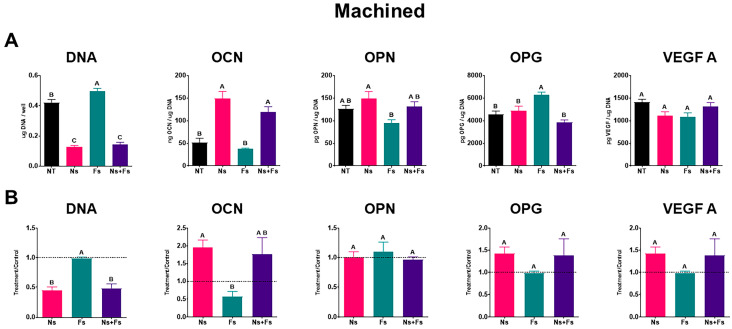
Cellular response of human bone marrow stromal cells to machined surfaces. Outcome measures include DNA, osteocalcin (OCN), osteopontin (OPN), osteoprotegerin (OPG), and vascular endothelial growth factor 165 (VEGF A). (**A**) Machined surface data were normalized to DNA. Values are means +/− SEM (*n* = 6) independent cultures per variable and were analyzed by ANOVA with Tukey post hoc testing. Bars that do not share a letter are statistically significant (*p* < 0.05). (**B**). Machined surface data were normalized to the NT group (treatment/control). T/C ratios (*n* = 3) were analyzed by ANOVA, followed by Tukey post hoc testing. Surface treatments included no laser treatment (NT), nanosecond laser (Ns), femtosecond laser (Fs), or nanosecond followed by femtosecond laser (Ns+Fs).

MSCs responded differentially to the surface topography and chemistry of the machined surfaces. The data shown in Figure 6A are from a single experiment, and the data in Figure 6B represent treatment/control ratios from three separate experiments, using the values on the NT surface as the control. The DNA content of the cultures was decreased on the surfaces treated with the nanosecond laser, irrespective of whether the surface was further modified with the femtosecond laser. In contrast, treatment with the femtolaser alone did not alter the DNA content compared to NT. Production of osteocalcin was increased in cultures grown on Ns and Ns+Fs, and the effect of the nanosecond laser was not reduced by Fs treatment. The surface properties did not affect the production of osteopontin, osteoprotegerin, and VEGF.

**Figure 7:**
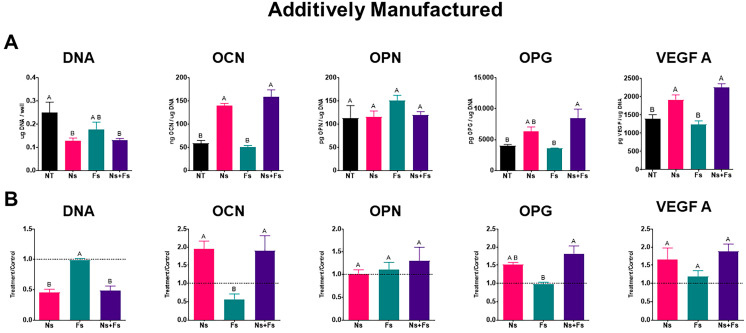
Cellular response of human bone marrow stromal cells to additively manufactured surfaces. Outcome measures include DNA, osteocalcin (OCN), osteopontin (OPN), osteoprotegerin (OPG), and vascular endothelial growth factor 165 (VEGF A). (**A**) Additively manufactured surface data were normalized to DNA. Values are means +/− SEM (*n* = 6) independent cultures per variable and were analyzed by ANOVA with Tukey post hoc testing. Bars that do not share a letter are statistically significant (*p* < 0.05). (**B**). Additively manufactured surface data were normalized to NT group (treatment/control). T/C ratios (*n* = 3) were analyzed by ANOVA followed by Tukey post hoc testing. Surface treatments included no laser treatment (NT), nanosecond laser (Ns), femtosecond laser (Fs), or nanosecond followed by femtosecond laser (Ns+Fs).

The response of MSCs to the additively manufactured disks was similar to their response to machined disks, but there were some important differences (Figure 7A,B). The DNA content of the cultures was reduced by Ns treatment. Similarly, osteocalcin production was markedly increased on the Ns and Ns+Fs surfaces. As noted for the machined disks, surface treatment did not affect osteopontin production. However, there was an increase in osteoprotegerin production on the Ns+Fs surfaces, and all treated surfaces had elevated VEGF compared to the NT control cultures.

**Figure 8:**
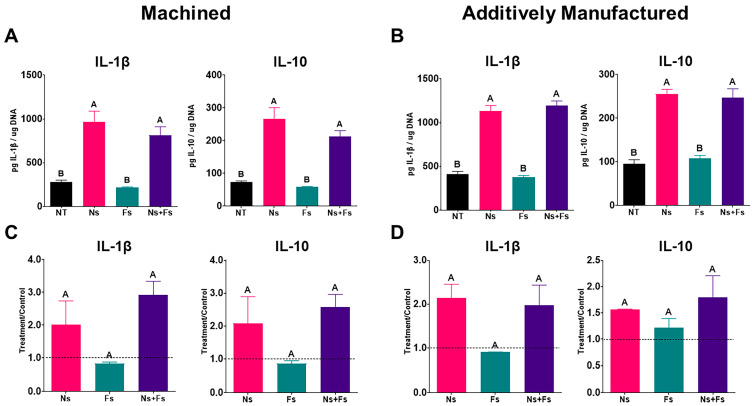
Immunomodulatory response of human bone marrow stromal cells to implant surfaces. Outcome measures include interleukin-1 beta (IL-1β) and interleukin-10 (IL-10). Machined surface data were normalized to DNA (**A**) and normalized to NT group (**B**). Additively manufactured surface data were normalized to DNA (**C**) and normalized to NT group (**D**). For (**A**) and (**B**), values are means +/− SEM (*n* = 6) independent cultures per variable and were analyzed by ANOVA with Tukey post hoc testing. Bars not sharing a letter are statistically significant (*p* < 0.05). For (**C**) and (**D**), T/C ratios (*n* = 3) were analyzed by ANOVA followed by Tukey post hoc testing. Surface treatments included no laser treatment (NT), nanosecond laser (Ns), femtosecond laser (Fs), or nanosecond followed by femtosecond laser (Ns+Fs).

Cytokine production was affected by the surface treatment, which reflected the fabrication method used to make the disks (Figure 8). MSCs grown on machined disks produced increased IL1β and IL10 on Ns and Ns+Fs surfaces compared to NT or Fs-only surfaces (Figure 8A). However, this effect of surface treatment was not evident when the treatment/control ratios for the three separate experiments were analyzed (Figure 8C). This was also the case for the production of IL1β and IL10 on the additively manufactured disks (Figure 8B,D).

## 4. Discussion

This study shows the importance of understanding implant surface properties in order to interpret cell culture results in designing a novel topography to enhance osseointegration. We found that the additive manufacturing of Ti6Al4V results in a surface that has different initial surface characteristics than conventionally machined Ti6Al4V, and these differences had small but significant effects of laser etching on the physical properties of the surface, as well as on the biological response of MSCs to the surface. The impact of the resulting implant surface properties (roughness, topographical features, chemistry, and wettability) is complex. They affect different stages of osteointegration from protein adsorption to cell adhesion and differentiation, bone formation, and bone modeling. We examined the culture’s response to Ti6Al4V substrates with these complex surface properties; therefore, a direct correlation between a specific parameter on a biological response was not possible. With this caveat in mind, however, our findings show a clear relationship between a laser-modified implant surface and a biological outcome and demonstrate how this is impacted by the manufacturing process used to fabricate the implant.

As noted by others, implants fabricated by additive manufacturing are rougher at the microscale than surfaces of implants fabricated by machining, and this was hypothesized to result in a more hydrophobic contact angle [31,32]. Alternative studies report that both machined and additively manufactured roughened Ti6Al4V surfaces are hydrophilic [33], as demonstrated here. Treatment of the surfaces with the femtosecond laser alone increased the hydrophilicity of the machined disks but reduced the hydrophilicity of the additively manufactured disks. This contradiction underscores the significance of the interaction between substrate morphology and treatment sequence. The initially smooth and uniform machined surfaces enabled the Fs treatment to reduce carbon contamination present on the surface while introducing minimal thermal alteration, resulting in a more hydrophilic state not typically found in Fs-treated additively manufactured surfaces. Treatment with the nanosecond laser alone caused the surfaces to become hydrophobic. Subsequent etching with the femtosecond laser restored hydrophilicity to the machined surfaces but further increased the hydrophobicity of the AM disks. The difference in wettability between the Fs and Ns+Fs groups is likely due to the large thermal effect of the Ns laser during the initial surface modification. The Fs laser, with ultra-short pulse durations and minimal heat output, produces finely structured nano-scale features, while the nanosecond laser’s thermal output leads to localized melting, resolidification, and micro-scale roughness. The effect of the reduction in C following the Fs laser ablation is less evident when performed on the significantly roughened Ns laser surface. Therefore, for both the machined and additively manufactured surfaces, there is no statistical difference between the Ns group and the Ns+Fs group.

Other factors may have contributed to the effects of laser treatment on hydrophobicity. Increased hydrophobicity has been seen where the creation of micro- and nanoscale roughness from nanosecond laser ablation and heat treatment resulted in superhydrophobic titanium surfaces. This phenomenon deviates from the Wenzel model’s prediction that roughness enhances wettability. This discrepancy is better explained by the Cassie–Baxter model, where air pockets trapped beneath the droplet, caused by roughened topography, lead to a higher contact angle despite increased surface roughness. In the present study, the Ns and Ns+Fs treatments yielded similar surface features, resulting in a metastable hydrophobic state. This model is also supported by the SEM micrographs of the nanosecond laser-treated surfaces, as micro-scale pores are seen [34,35]. This has been attributed to the high thermal output of the nanosecond laser ablation, which alters the properties of the titanium alloy surface by increasing the thickness of the titanium oxide layer [36,37].

Treatment with the femtosecond laser had opposite effects between machined and additively manufactured surfaces, indicating that the manufacturing process affects how the laser interacts with the surface. The thickening of the Ti oxide layer on the machined surfaces, as demonstrated by the increased presence of oxygen and titanium on the surfaces of the Ns and Ns+Fs groups, was accompanied by a decrease in carbon, which was also observed in the additively manufactured Ns+Fs disks. The presence of the oxide layer is vital for implant success. It reduces the release of metallic ions that can negatively impact reactions in nearby tissues and prevents corrosion of the implant by host tissue fluids. Studies show that it enhances osteogenic activity in vitro and improves bone-to-implant contact in vivo [38,39]. Laser treatment, especially the nanosecond laser treatment tested here, can increase TiO_2_ thickness due to localized heating and subsequent reoxidation during cooling. The machined surfaces are more uniform. In contrast, the additively manufactured surfaces, with higher roughness, may lead to uneven oxide layer formation or even partial masking of oxide layer growth in regions with smoother baseline morphology. Although we did not measure oxide thickness via cross-sectional methods (such as TEM), the increase in oxygen signal intensity via XPS suggests greater oxide layer development for machined surfaces. The change in the oxygen on the surface, with a probable change in the TiO_2_ thickness by the laser treatment, affects the biological response.

The decrease in elemental carbon was likely due to the reduced hydrocarbons on the surface after cleaning with the femtosecond laser. The hydrocarbons present on the surfaces are likely due to atmospheric exposure during handling and loading, as carbon has been well-documented on the surface of titanium implants [40,41]. Care must be taken to minimize this contamination, such as by placing the implant in an aqueous environment or plasma treatment. Previous studies have shown that reduced hydrocarbon adsorption increases protein adsorption on the surface, particularly fibronectin, and thus improves osteogenic cell attachment and growth, and ultimately osseointegration [42,43]. For both manufacturing techniques, the increased elemental aluminum detected after the Fs treatments reflects the localized enrichment and redistribution that can arise due to the different laser ablation effects. Although the effects on biological response caused by this cannot be isolated from topographical changes, these laser-induced surface chemistry changes likely had a combined effect.

The physical changes in the surface topography caused by the nanosecond laser resulted in significant increases in Sa and Sz roughness parameters for Ns and Ns+Fs, regardless of fabrication method. The increase in roughness was due primarily to etching by the nanosecond laser. This is a direct result of the longer pulse duration producing greater thermal output and subsequent resolidification. In contrast, the ultrashort pulse duration of the femtosecond laser superimposed a nanotopography that was ridge-like, with a ridge occurring approximately every 250–500 nm. The Ns etching, we believe, is the sole reason for the resulting morphology and increase in roughness. The shorter pulse duration of the Fs laser shows this same effect, which does not significantly change the roughness parameters measured. This effect was present on machined and additively manufactured surfaces, although it was less pronounced on the AM background.

During normal bone remodeling, osteoprogenitor cells migrate into pits created by osteoclasts, characterized as being roughly 30–100 μm in width and 2–10 μm in depth [44,45]. Studies show that MSCs differentiate into osteoblasts when cultured on Ti or Ti6Al4V surfaces with topography that features microscale structures comparable to those of an osteoclast resorption pit [14], and this differentiation is further enhanced by the presence of nanoscale features with low skewness and kurtosis. However, to date, no surface has been reported that recapitulates explicitly the nanoscale topographic features of an osteoclast resorption pit. High-resolution transmission electron microscopy reveals that the base of the osteoclast resorption pits exhibits nanoscale roughness, similar to the physical structures generated by treating Ti6Al4V disks with Ns and Ns+Fs laser etching. We believe this structure closely mimics the morphology of an osteoclast resorption pit and has been shown to push MSCs to an osteoblast phenotype. Surfaces with these characteristics exhibit greater bone-to-implant contact and stability in vivo, thereby strengthening the argument that Ns-treated surfaces can enhance the osseointegration of implants [46].

MSCs grown on both machined and additively manufactured disks treated with Ns or Ns+Fs exhibited reduced DNA and increased osteocalcin per DNA, which is a consistent observation for MSCs undergoing osteoblast differentiation in vitro [47], and is correlated with improved osseointegration in vivo [48]. Osteopontin, which is produced by osteoblasts as a component of the extracellular matrix [49], was not sensitive to Ns or Fs treatment of the surface. Osteoprotegerin, which is produced by osteoblasts to regulate osteoclast activity [50], was elevated in cultures grown on Ns+Fs surfaces, but the effects were not significant across all experiments. This was also the case for VEGF, which promotes vascularization. These results support the hypothesis that the nanoscale modification of the Ti6Al4V surfaces had a specific regulatory effect, initiating osteoblast differentiation.

These experiments were conducted using growth media, eliminating artifacts due to additives in osteogenic media that stimulate osteoblast differentiation [51,52]. MSCs were cultured for 7 days without the use of osteogenic media, a key factor in interpreting our results. Osteogenic media is high in calcium ions, and along with the addition of beta-glycerol phosphate, can cause dystrophic calcification to occur in MSC cultures [53]. OM is also supplemented with ascorbic acid and dexamethasone, components that enable the activation of Runx2 and β-catenin/Wnt pathways that have been shown to induce osteoblastic differentiation [51]. The stimulatory effect on osteocalcin observed in the present study was evident by 7 days in growth media, whereas cells grown in osteogenic media on TCPS do not exhibit an osteoblast phenotype for 14–21 days [54]. By culturing the MSCs on the Ns and Ns+Fs surfaces in normal growth media, the results suggest that a push toward an osteoblast phenotype can be more accurately attributed to surface-dependent effects.

MSCs grown on Ns and Ns+Fs surfaces exhibited elevated levels of cytokines, including the proinflammatory IL-1β and the anti-inflammatory IL-10. This conflicts with many findings looking at Ti alloy surfaces modified by grit blasting and acid etching, which show that MSC expression of proinflammatory interleukins (IL-1α, IL-1β, and IL-6) decreases, while anti-inflammatory interleukin (IL-1RN, IL-4, and IL-10) expression increases in many cases [55]. These data capture a snapshot at a single time point, indicating the dynamic nature of the inflammatory response. The co-expression of IL-1β and IL-10 may reflect the early initiation and regulation of an immune response. While IL-1β helps to activate inflammatory signaling and promote MSC migration, IL-10 acts to dampen inflammation and facilitate resolution. This biphasic cytokine profile has been associated with pro-regenerative environments in wound healing models and also highlights the diverse results that can come from culture conditions and cells studied [56]. However, a balanced immune response to injury can be favorable for overall patient healing, as the initiation of the immune response by IL-1β can thereafter be controlled by the elevated IL-10 [57,58]. Further study is needed to understand the surface-mediated immune effect.

## 5. Conclusions

The results clearly indicate that MSCs cultured on Ti6Al4V surfaces treated using nano-laser followed by femto-laser ablation differentiate into osteoblasts. These findings suggest that these nanoscale surface modifications are an effective approach to improving implant integration and patient outcomes. This effect was induced by mimicking the roughened morphology of osteoclast resorption pits found in bone, supported by the increased production of osteocalcin. These results also provide insight into how implant manufacturing methods influence the effectiveness of laser treatments in achieving specific surface topographical features, resulting in desired biological outcomes. The present study indicates the advantage of laser treatment in generating surfaces with a controlled topography to improve a biological response, enhancing the osseointegration process and implant success.

## Data Availability

The raw data supporting the conclusions of this article will be made available by the corresponding author upon reasonable request.

## Supplementary Materials

The following supporting information can be downloaded at: https://www.mdpi.com/article/10.3390/biomimetics10080507/s1, Figure S1: Surface profilometry obtained from confocal LSM microscopy demonstrated the topographical differences due to various laser ablation treatments on machined surfaces. Z-stack images to create profiles had a 3-pixel Gaussian filter and a 20 μm high-pass filter applied. Surface treatments included no laser treatment (NT), nanosecond laser (Ns), femtosecond laser (Fs), or nanosecond followed by femtosecond laser (Ns+Fs); Figure S2: Surface roughness parameters demonstrating the topographical differences due to various laser ablation treatments on machined surfaces. Z-stack images measured had a 3-pixel Gaussian filter and a 20 μm high-pass filter applied. Surface treatments included no laser treatment (NT), nanosecond laser (Ns), femtosecond laser (Fs), or nanosecond followed by femtosecond laser (Ns+Fs). Groups not sharing a letter are significantly different (*p* < 0.05). Data are means +/− SEM (*n* = 6).

## Abbreviations

The following abbreviations are used in this manuscript:

Ti6Al4V	Titanium–aluminum–vanadium
MSCs	Bone marrow stromal cells
M	Machined
AM	Additively manufactured
NT	No laser treatment
Ns	Nanosecond laser
Fs	Femtosecond laser
Ns+Fs	Nanosecond followed by femtosecond laser
XPS	X-ray photoelectron spectroscopy
LSM	laser scanning microscopy
SEM	scanning electron microscopy
TCPS	tissue culture polystyrene
OCN	Osteocalcin
OPN	Osteopontin
OPG	Osteoprotegerin
VEGF	vascular endothelial growth factor 165
IL-1β	interleukin-1 beta
IL-10	interleukin-10
LIPSS	laser-induced periodic surface structures
GM	growth media
OM	osteogenic media
DMLS	direct metal laser sintering
HIP	hot isostatic pressing
T	laser-treated
L	Lighting
ΔE	change in color variable
Sa	arithmetic mean deviation
Sz	maximum peak to valley distance
ELISA	enzyme-linked immunosorbent assay
ANOVA	one-way analysis of variance
T/C	treatment/control ratios
