# Supplementary material for: Titanium–Aluminum–Vanadium Surfaces Generated Using Sequential Nanosecond and Femtosecond Laser Etching Provide Osteogenic Nanotopography on Additively Manufactured Implants
Source: Biomimetics (Basel). 2025 Aug 4;10(8):507. doi: 10.3390/biomimetics10080507 (PMC12383309; doi:10.3390/biomimetics10080507)
Supplement: Supplementary file 1 [file biomimetics-10-00507-s001.zip › biomimetics-3732443-supplementary.pdf]

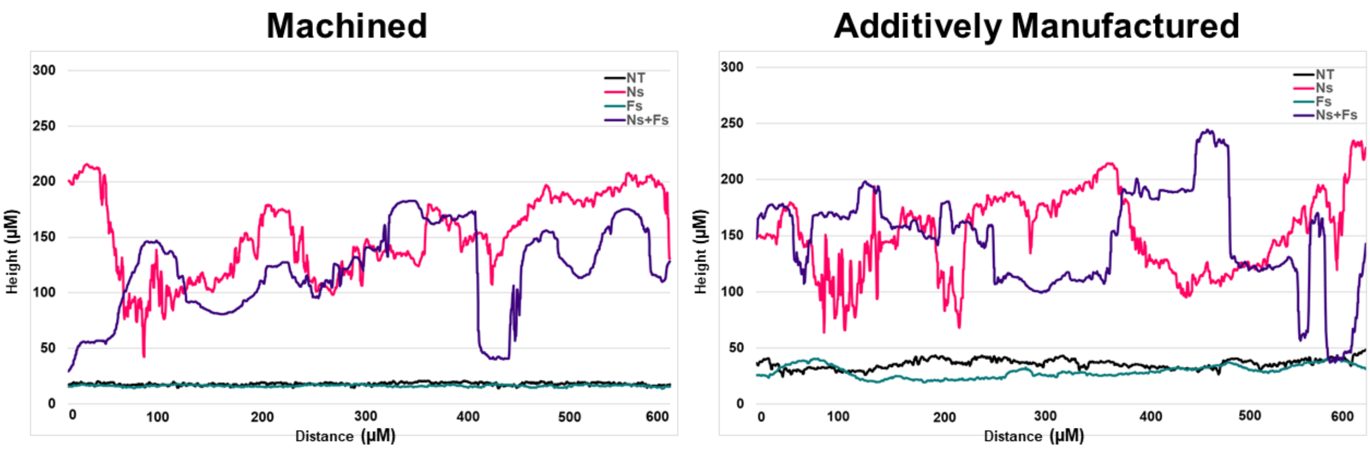


**Figure S1.** Surface profilometry obtained from confocal LSM microscopy demonstrated the topographical differences due to various laser ablation treatments on machined surfaces. Z-stack images to create profiles had a 3-pixel Gaussian filter and a 20µm high-pass filter applied. Surface treatments included no laser treatment (NT), nanosecond laser (Ns), femtosecond laser (Fs), or nanosecond followed by femtosecond laser (Ns+Fs).

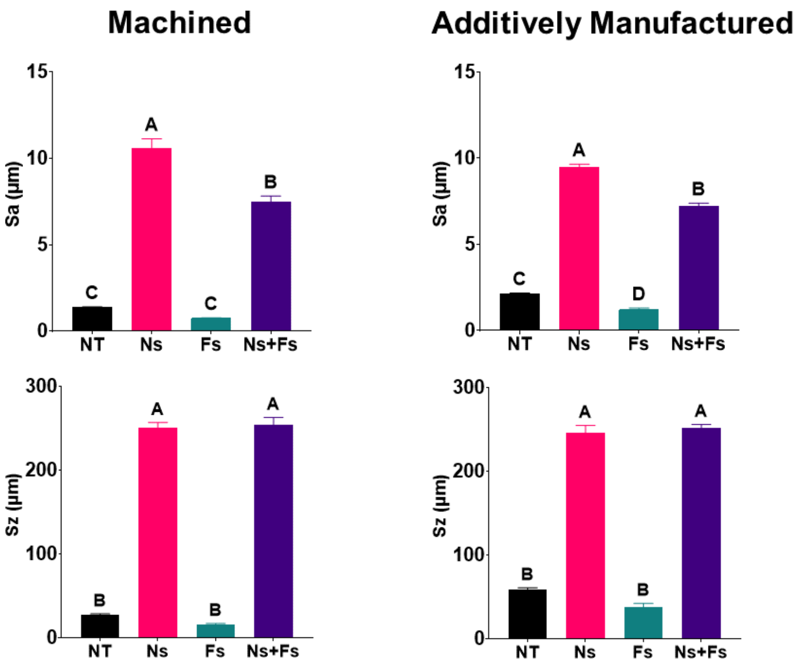


**Figure S2.** Surface roughness parameters demonstrating the topographical differences due to various laser ablation treatments on machined surfaces. Z-stack images measured had a 3-pixel Gaussian filter and a 20µm high-pass filter applied. Surface treatments included no laser treatment (NT), nanosecond laser (Ns), femtosecond laser (Fs), or nanosecond followed by femtosecond laser (Ns+Fs). Groups not sharing a letter are significantly different ($p < 0.05$). Data are means +/− SEM ($n = 6$).
